# Frequency of SCID‐5‐RV psychiatric symptoms in individuals with Alzheimer's disease and related dementias vs. older adults

**DOI:** 10.1002/alz70857_105513

**Published:** 2025-12-25

**Authors:** Megan S Barker, Alyssa N. De Vito, Zachary J. Kunicki, Masood Manoochehri, Claudine K. Narayan, Juan Pabloga Caji, Colin Stein, Nicole Belanger, Edward D. Huey

**Affiliations:** ^1^ Brown University, Providence, RI, USA; ^2^ Butler Hospital Memory and Aging Program, Providence, RI, USA; ^3^ Brown University Medical School, Providence, RI, USA; ^4^ Alpert Medical School of Brown University, Providence, RI, USA; ^5^ Department of Psychiatry and Human Behavior, Alpert Medical School of Brown University, Providence, RI, USA

## Abstract

**Background:**

Neuropsychiatric symptoms (NPS) in Alzheimer's disease and related dementias (AD/ADRD) greatly increase mortality and caregiver burden. However, examinations of NPS have been limited by a lack of global, comprehensive assessments of psychiatric symptomatology in AD/ADRD. Comparing symptoms in AD/ADRD with older adults without dementia is of key importance for distinguishing pathological from normal aging. Here, we examine data collected using the Structured Clinical Interview for DSM‐5‐Research Version (SCID‐5‐RV), which is a gold‐standard measure of psychiatric symptoms, and compare frequencies in individuals with AD/ADRD to a control group of older adults without dementia.

**Method:**

The study sample comprised 237 participants, including 49 older adults without dementia (M age = 63.2±13.1), and 188 individuals with AD/ADRD (M age=69.9±10.1; 78 Alzheimer's disease dementia, 110 other dementia). Item‐level data on the SCID‐5‐RV was collected, including all symptom questions (skip rules were not used). Prevalence of symptom endorsement was compared between the AD/ADRD and control groups, using Pearson's Chi‐squared test or Fisher's exact test.

**Result:**

Compared to older adults without dementia, the AD/ADRD group endorsed more frequent sleep problems, including fatigue, excessive sleepiness, daytime napping, prolonged nonrestorative sleep, and difficulty waking (all *p* < .05). Depression‐related symptoms were among the most endorsed within both groups, but the ADRD group had more frequent depressed mood, anhedonia, avolition, feelings of guilt (all *p* < .05). Notably, suicidality was present in 10% of the AD/ADRD group and 0% of controls (*p* < .05). While the AD/ADRD group reported more anxiety in terms of worry, irritability, restlessness, and muscle tension, controls exhibited more panic and phobia symptoms (all *p* < .05). The AD/ADRD group also reported more psychomotor agitation and verbal aggression (*p* < .05), and, unsurprisingly, more difficulties thinking and concentrating (*p* < .05). Overall, this points to psychiatric phenotypes in AD/ADRD

**Conclusion:**

The results highlight important differences between reported psychiatric symptoms in those with AD/ADRD compared to unimpaired older adults. These findings can be used to develop measures that capture relevant neuropsychiatric constructs for use in neurodegenerative populations, and suggest specific symptoms that comprise psychiatric phenotypes that might point to pathological vs. normal aging.

